# Dl-3-N-Butylphthalide Promotes Angiogenesis in an Optimized Model of Transient Ischemic Attack in C57BL/6 Mice

**DOI:** 10.3389/fphar.2021.751397

**Published:** 2021-09-29

**Authors:** Jiahui Wang, Yanyan Li, Haihan Yu, Gaigai Li, Shuang Bai, Shiling Chen, Ping Zhang, Zhouping Tang

**Affiliations:** Department of Neurology, Tongji Hospital, Tongji Medical College, Huazhong University of Science and Technology, Wuhan, China

**Keywords:** model, C57BL/6 mice, transient ischemic attack, middle cerebral artery occlusion, Dl-3-n-butylphthalide, angiogenesis

## Abstract

Transient ischemic attack (TIA) has been widely regarded as a clinical entity. Even though magnetic resonance imaging (MRI) results of TIA patients are negative, potential neurovascular damage might be present, and may account for long-term cognitive impairment. Animal models that simulate human diseases are essential tools for in-depth study of TIA. Previous studies have clarified that Dl-3-N-butylphthalide (NBP) promotes angiogenesis after stroke. However, the effects of NBP on TIA remain unknown. This study aims to develop an optimized TIA model in C57BL/6 mice to explore the microscopic evidence of ischemic injury after TIA, and investigate the therapeutic effects of NBP on TIA. C57BL/6 mice underwent varying durations (7, 8, 9 or 10 min) of middle cerebral artery occlusion (MCAO). Cerebral artery occlusion and reperfusion were assessed by laser speckle contrast imaging. TIA and ischemic stroke were distinguished by neurological testing and MRI examination at 24 h post-operation. Neuronal apoptosis was examined by TUNEL staining. Images of submicron cerebrovascular networks were obtained *via* micro-optical sectioning tomography. Subsequently, the mice were randomly assigned to a sham-operated group, a vehicle-treated TIA group or an NBP-treated TIA group. Vascular density was determined by immunofluorescent staining and fluorescein isothiocyanate method, and the expression of angiogenic growth factors were detected by western blot analysis. We found that an 8-min or shorter period of ischemia induced neither permanent neurological deficits nor MRI detectable brain lesions in C57BL/6 mice, but histologically caused neuronal apoptosis and cerebral vasculature abnormalities. NBP treatment increased the number of CD31^+^ microvessels and perfused microvessels after TIA. NBP also up-regulated the expression of VEGF, Ang-1 and Ang-2 and improved the cerebrovascular network. In conclusion, 8 min or shorter cerebral ischemia induced by the suture MCAO method is an appropriate TIA model in C57BL/6 mice, which conforms to the definition of human TIA, but causes microscopic neurovascular impairment. NBP treatment increased the expression of angiogenic growth factors, promoted angiogenesis and improved cerebral microvessels after TIA. Our study provides new insights on the pathogenesis and potential treatments of TIA.

## Introduction

Transient ischemic attack (TIA) is a brief episode of neurological dysfunction resulting from focal brain, spinal cord or retinal ischemia, without evidence of acute cerebral infarction (Easton et al., 2009). According to this histology-based definition, the absence of ischemic high signal on diffusion weighted imaging (DWI) sequence of magnetic resonance imaging (MRI) is commonly used as one of the diagnostic criteria of TIA. Population-based studies indicate that the age-standardized morbidity of TIA in Europe is 28–59/100,000/year (Bejot et al., 2016). Furthermore, in the United States, approximately 240,000 TIAs are diagnosed each year (Johnston et al., 2007). In China, the age-standardized incidence of TIA is 2.27%, but only 16.0% of total TIAs are diagnosed (Wang et al., 2015). Recent studies have found that TIA is an important warning signal of stroke, and 15–30% of patients with ischemic stroke had a history of TIA (Rothwell and Warlow, 2005). Moreover, the risk of stroke within 90 days after TIA is 10–20%, of which 50% occur within the first 2 days (Hill et al., 2004; Johnston et al., 2007). In addition, patients with TIA may experience brain atrophy and cognitive decline, which is a key risk factor for dementia (Sivakumar et al., 2014; van Rooij et al., 2014; Bivard et al., 2018). However, the pathogenesis and pathophysiological changes associated with TIA have not been fully clarified. Therefore, it is urgently necessary to develop an appropriate animal model of TIA to investigate neurovascular changes and interventions for TIA.

The intraluminal suture middle cerebral artery occlusion (MCAO) method is minimally invasive and easy to perform, with the ability to precisely control the ischemia-reperfusion process, and thus is a preferable method for TIA modeling. Based on this method, Durukan (Durukan et al., 2017) and Pedrono (Pedrono et al., 2010) established TIA models in Wistar rats and NMRI mice, respectively, by controlling the duration of ischemia. They found that in both species, no cerebral infarction confirmed by MRI occurred when the occlusion lasted no more than 10 min. However, a 10-min period of MCAO in SD rats produced visible brain injury on MRI (33% of the rats) (Fan et al., 2017). In addition, 41% of the ICR-CD1 mice showed striatal lesions on MRI after 10 min of ischemia (Berthet et al., 2011). Taken together, the tolerance of different species to cerebral ischemia is diverse, and the above experimental results cannot be directly applied to other animal strains.

The C57BL/6 mouse is the most widely used background strain of transgenic mice, in which the global ischemia model shows high reproducibility and consistent neuronal damage (Yonekura et al., 2004). Additionally, compared to other strains, the C57BL/6 mouse is more vulnerable to focal cerebral ischemia, which is attributed to their cerebral vascular anatomy (the lack of one or both posterior communicating arteries) (Mccoll et al., 2004). Here, we aim to develop a TIA model in C57BL/6 mice by exploring the ischemia duration based on the intraluminal suture MCAO method. To realize the relevance to clinical TIA, three criteria of TIA model were proposed (Pedrono et al., 2010; Durukan et al., 2017): 1) objective evidence of ischemia and reperfusion, 2) no permanent neurological deficit, and 3) no acute cerebral infarction. In this study, laser speckle contrast imaging (LSCI) was used during surgery to evaluate cerebral arteries occlusion and reperfusion. Neurological testing and MRI examination [T2-weighted imaging (T2WI) and DWI] were performed at 24 h after reperfusion to determine the absence of neurological deficit and cerebral infarction. After the detection of the critical ischemia duration that limits the outcome to TIA rather than stroke, histopathological staining was used to identify the microscopic evidences of ischemic injury after TIA. Micro-optical sectioning tomography (MOST), which can perform three-dimensional (3D) imaging of the whole brain at submicron resolution and provide precise visualization of the neurovascular network, has been used to reconstruct and describe the entire cerebral vascular system of mice (Xiong et al., 2017). Herein, we also employed MOST to detect changes in the cerebrovascular system after TIA at submicron resolution.

Dl-3-N-butylphthalide (NBP) is a small molecule drug that was originally harvested from celery seeds before being independently developed in China for the treatment of ischemic stroke. It acts by reducing cerebral ischemic injury through multiple mechanisms including anti-inflammatory, antioxidant, anti-apoptosis and anti-thrombosis (Xu and Feng, 2000; Chang and Wang, 2003; Li et al., 2018; Qin et al., 2019; Yang et al., 2019). Previous reports showed that NBP can protect brain microvascular endothelial cells against oxygen-glucose deprivation induced damage by up-regulating the expression of HIF-1α (Yang et al., 2012). NBP can also increase the expression of angiogenic growth factors and promote angiogenesis in stroke models (Liu et al., 2007; Liao et al., 2009; Zhou PT. et al., 2019). Moreover, NBP can increase regional cerebral blood flow (CBF) after cerebral ischemia (Yan et al., 1998). However, the effects of NBP on TIA remain unknown. Therefore, this study further explored the effects of NBP on angiogenesis after TIA and possible mechanisms underlying these effects.

## Materials and Methods

### Animals

Adult male C57BL/6 mice (8–10 weeks, 22–25 g) were provided by the Animal Center of Tongji Hospital of Tongji Medical College of Huazhong University of Science and Technology (Wuhan, China). The animals were housed in a controlled environment at a temperature of 18–22°C under a 12-h light/dark cycle, with free access to standard food and water. All animal experiments were performed in accordance with the National Institutes of Health Guidelines for the Care and Use of Laboratory Animals. The study protocol was approved by the Experimental Animal Ethics Committee of Huazhong University of Science and Technology (Wuhan, China). We made all attempts to minimize suffering of experiment animals and limit the number of animals used. All surgeries and evaluations were performed by investigators blinded to the experimental condition of each animal.

### Study Design and Experimental Grouping

**FIGURE 1:**
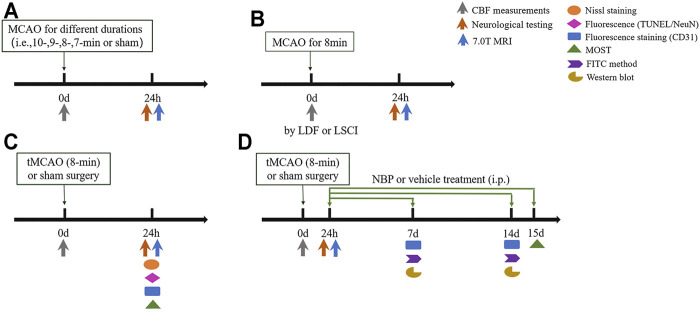
Experimental protocol. Mice were divided into 4 cohorts: **(A)** Experiment I, determination of the optimal ischemia duration to induce transient ischemic attack (TIA). **(B)** Experiment II, comparation of the methods for cerebral blood flow (CBF) monitoring. **(C)** Experiment III, investigation of the histopathological changes induced by TIA. **(D)** Experiment IV, exploration of the therapeutic effects of Dl-3-N-butylphthalide (NBP) on TIA.

The mice were randomly assigned to four separate experiments designed as follows (Figure 1).

#### Experiment I

In our preliminary experiment, transient ischemia of 5, 7, and 10 min were tested. The results showed that no mice in the 5- or 7-min ischemia groups had MRI detectable lesions, while animals in the 10-min group developed infarction (Supplementary Figure 1), indicating that the infarct threshold (the minimum ischemia duration inducing cerebral infarction) in C57BL/6 mice was between 7 min and 10 min. Therefore, to further determine the critical ischemia duration limiting the outcome to TIA rather than stroke, the mice were randomly divided into five groups: 1) Groups 1–4 were subjected to 10-, 9-, 8-, or 7-min MCAO, respectively (i.e., the suture occluded the origin of the middle cerebral artery (MCA) for 10, 9, 8, 7 min, respectively), 2) Group 5 was a control group comprised of sham-operated animals. In each group, CBF was monitored by LSCI, and neurological testing and MRI examination were performed at 24 h after surgery.

#### Experiment II

Laser Doppler flowmeter (LDF) and LSCI are currently the most commonly used methods for monitoring CBF. LSCI can simultaneously measure CBF across the surface of the entire cortex, while LDF can only perform single-point measurements. To compare these methods of monitoring CBF, the mice were randomly divided into 1) LDF-monitored group or 2) LSCI-monitored group, in which LDF or LSCI was used to monitor CBF during TIA surgery.

#### Experiment III

After determining the optimal duration of ischemia to induce TIA, Experiment III was designed to investigate the histopathological changes induced by TIA. Mice were randomly assigned to 1) control group (sham-operated animals) or 2) TIA group. Neuronal injury was examined by Nissl and TUNEL staining, and cerebrovascular changes were assessed *via* immunofluorescence and MOST analysis at 24 h post-surgery.

#### Experiment IV

To explore the therapeutic effects of NBP on TIA, the mice were randomly divided into three groups: 1) sham group (sham-operated animals injected with saline), 2) vehicle-treated group (TIA mice injected with saline), or 3) NBP-treated group (TIA mice treated with NBP). NBP concentrated (25 mg/5 ml) injection solution was obtained from Shijiazhuang Pharmaceutical Group dl-3-butylphthalide Pharmaceutical Co. LTD. (Shijiazhuang, China). NBP (30 mg/kg body weight, i.e., 0.06 ml/10g) or equal volume of vehicle (normal saline) was administered intraperitoneally once a day from 1 day after MCAO to the day of execution. The dose and frequency of NBP was chosen in reference to previous studies reporting neuroprotective effects of this dosage on cerebral ischemia (Chang and Wang, 2003; Yang et al., 2015; Xu et al., 2017). The establishment and verification of the TIA model was in accordance with Experiment I, mainly as CBF measurements with LDF during the operation, and neurological testing and MRI examination at 24 h after surgery. Mice in each group were randomly selected for immunofluorescence analysis, fluorescein isothiocyanate (FITC) method, western blot analysis or fluorescence MOST (fMOST) analysis at 7, 14 or 15 days after MCAO.

### Transient Focal Cerebral Ischemia

Transient focal cerebral ischemia was induced by using the suture MCAO model (Koizumi et al., 1986; Longa et al., 1989). Briefly, mice were anesthetized with isoflurane (2.5% induction and 1.5% maintenance in 100% oxygen). The right common carotid artery, the right external carotid artery and the right internal carotid artery were exposed through a midline neck incision. After that, a silicone rubber-coated monofilament (L1800; Guangzhou Jialing Biotech Co. LTD., Guangzhou, China) was inserted from the external carotid artery to internal carotid artery until a slight resistance was felt (about 9–10 mm), indicating that the suture had occluded the origin of the MCA. Reperfusion was initiated by withdrawing the suture after specified duration of MCAO. Sham-operated animals experienced the same operational procedures except the insertion of suture. The rectal temperature of the mice was maintained at 37.0 ± 0.5°C throughout the operation with a heating blanket.

### Cerebral Blood Flow Measurement by Laser Speckle Contrast Imaging

LSCI was used to visualize regional CBF. Under isoflurane anesthesia, the head of the mouse was fixed on a stereotaxic apparatus. After 75% alcohol disinfection, the scalp was incised longitudinally to fully expose the skull, which was swabbed with a saline cotton ball to keep it moist. Subsequently, the mouse was placed under the LSCI system (National Laboratory for Optoelectronics, Wuhan, China) to observe its CBF images. After adjusting the focal length, the white light source of the microscope was used to select the imaging field, which was positioned to include both hemispheres, and then a 660-nm diode laser was used to illuminate the skull surface in a diffuse and uniform manner. The scattered light signals in the observation area were collected by a 12-bit CCD camera (PixelFly VGA, PCO Computer, Germany) connected to a stereoscopic microscope (SZ61, OLYMPUS, Japan), with a resolution of 640 × 480 pixels. Shutter speed was set to an exposure time of 20 ms. The raw images were directly converted into blood-flow velocity information through real-time blood-flow arithmetic processing, and images were continuously acquired at a rate of 1 frame per second for 60 frames. Data analysis was performed by converting the blood-flow velocity information into a two-dimensional blood flow map by computer algorithm, and the partial images were processed by inverting color with the system’s own image software. Because the spatial distribution of ischemia was slightly different among animals, a wide region of interest (ROI; a circle with 2.5-mm diameter) within the MCA blood supply area (also corresponding to the LDF measurement region) was selected to measure CBF. CBF images were collected at pre-operation (baseline), occlusion (MCAO), and at 15 and 30 min of reperfusion.

### Cerebral Blood Flow Measurement by Laser Doppler Flowmeter

Regional CBF was also measured by LDF (Moor Instruments, Axminster, Devon, United Kingdom). A flexible fiber-optic probe was fixed on the right parietal bone surface (2 mm posterior and 5 mm lateral to the bregma) with tissue adhesive to monitor the CBF of the MCA blood supply area. Similarly, CBF values were recorded at baseline, MCAO and at 10, 20, and 30 min of reperfusion.

A successful occlusion was confirmed by a >75% decrease in CBF. All animals with unsuccessful occlusion or inadequate reperfusion were excluded (Criterion 1).

### Neurological Evaluation

A six-point sensorimotor scale (Tatlisumak et al., 1998) was used to assess neurological status of the animals at 24 h after MCAO. The scoring criteria were as follows: a score of 0 indicated no neurological deficit, a score of 1 indicated failure to extend left forepaw fully, a score of 2 indicated a walking gait circling to the left, a score of 3 indicated falling to the left, a score of 4 indicated no spontaneous walking with a depressed level of consciousness, and a score of 5 indicated death. No neurological deficit was a requirement for the TIA model (Criterion 2).

### 7.0 T Magnetic Resonance Imaging

After the neurological evaluation (at 24 h and 7 days after MCAO), mice were anesthetized for a coronal brain MRI scanning, including T2WI sequence and DWI sequence. This was performed by using a 7.0 Tesla scanner (20 cm, Bruker Biospect 7.0 T, Germany). A linear birdcage radio frequency coil with an inner diameter of 19 mm was used. T2WI was obtained using a T2-weighted rapid acquisition with relaxation enhancement (RARE) sequence (TR/TE = 3000/22.5 ms, slice thickness = 0.8 mm, spatial resolution = 0.078 mm). Diffusion-weighted images were acquired by using an echo planar imaging (EPI) sequence with b value of 500 s/mm^2^ (TR/TE = 3000.00/30.00 ms, slice thickness = 0.8 mm, spatial resolution = 0.156 mm). Both DWI and T2WI sequences had 12 slices covering the entire brain. During the MRI examination, the body temperature of the mouse was maintained at 37°C with the thermostatic water heating system configured by the scanner. Images were evaluated visually to detect any signal-enhancement regions that indicated ischemic lesions. Ischemic lesions on T2WI scans were manually outlined in each slice using Image J software (National Institutes of Health, MD, United States). The lesion areas were accumulated and multiplied by the slice thickness to obtain lesion volume, and the ratio of the lesion volume to the volume of the contralateral hemisphere was recorded. MRI scans indicating no infarct lesions fulfilled Criterion 3 of TIA model.

### Preparation for Histopathology

The mice were deeply anesthetized with pentobarbital sodium (50 mg/kg, i.p.) and then transcardially perfused with normal saline and 4% paraformaldehyde (PFA). The brains were harvested and post-fixed in 4% PFA overnight, followed by gradient dehydration with 20 and 30% sucrose. The brain tissues were cut into 15 µm thick coronal sections (CM 1950, Leica, Germany) and mounted onto glass slides for subsequent staining.

### Nissl Staining

To evaluate neuronal damage, Nissl staining was performed. In short, the brain tissues were embedded in paraffin and sliced into 5 µm thick coronal sections. The sections were dewaxed with xylene and rehydrated in ethanol. Then, the sections were immersed in Nissl staining solution (Servicebio, China) for 5 min at room temperature. This was followed by distilled water rinse to remove excess staining solution. Next, the sections were differentiated with 0.1% glacial acetic acid, and cleared with xylene. After applying a coverslip, the sections were analyzed with a light microscope (BX-51, OLYMPUS, Tokyo, Japan).

### TUNEL Fluorescence

Apoptosis of neurons was assessed quantitatively by co-labeling with NeuN and TUNEL. TUNEL staining was performed using the *in situ* cell death detection kit (Fluorescein; Roche, Switzerland) in accordance with the manufacturer’s instructions, and then co-stained to label neurons. Briefly, the sections were rinsed in phosphate buffered saline (PBS) and then placed in 0.1 mol/L citrate buffer (PH 6.0) in which they were exposed to microwave irradiation for 1 min. After blocking with 10% bovine serum albumin (BSA) containing 0.25% Triton X-100 for 30 min at room temperature, the sections were incubated in TUNEL reaction mixture for 1 h at 37°C in the dark. For the negative controls, label solution without terminal transferase was used instead of TUNEL reaction mixture. Subsequently, the sections were incubated with primary antibody of rabbit anti-NeuN (1:200; Cell Signaling Technology, United States) at 4°C overnight. Thereafter the sections were incubated with secondary antibody of Cy3 conjugated goat anti rabbit IgG (1:200; Servicebio, China) for 1 h at room temperature and then counterstained with 4’,6-diamidino-2-phenylindole (DAPI; Servicebio, China) for 5 min. The sections were observed under a fluorescent microscope with a 20× or 40× objective lens (BX-51, OLYMPUS, Tokyo, Japan). Three sections (150 μm before, center, and 150 μm after ischemia center) (Zhou PT. et al., 2019) for each mouse were selected for tissue analysis. Each section was analyzed by randomly selecting at least three microscopic fields in the area of interest of the ipsilateral hemisphere (the gray area in Supplementary Figure 2). The number of TUNEL/NeuN double-positive cells and DAPI-labeled cells in each slice were manually counted by evaluators blinded to the experimental grouping. The percentage of double-positive cells relative to the total number of cells labeled with DAPI was calculated.

### CD31 Immunofluorescent Staining

To investigate vascular changes, immunofluorescent staining for CD31 was conducted. In brief, the sections were placed in EDTA repair solution (PH 8.0) then exposed to microwave irradiation for 5 min. After cooling for about 1 h, the sections were blocked with 10% BSA for 1 h at room temperature. Next, the sections were incubated with primary antibody of rabbit anti-CD31 (1:100; Cell Signaling Technology, United States) at 4°C overnight, followed by incubation with the corresponding secondary antibody and then with DAPI. The primary antibody was replaced by PBS for the negative controls. Three sections from each mouse were analyzed. For each section, four microscopic fields in the area of interest were randomly selected and observed under a fluorescent microscope at 200× magnification. The number of CD31^+^ microvessels in each slice was manually calculated by an investigator blinded to the experimental groups and the values from 4 random fields were averaged.

### Fluorescein Isothiocyanate Method

FITC method was used to detect the changes in the density of perfused vessels (functional vessels). FITC is a fluorescein derivative that can covalently bind to vascular endothelial cells and accumulate in the nucleus under alkaline pH conditions to mark cerebral perfused vessels (Miyata and Morita, 2011; Yu et al., 2014). After anesthesia with pentobarbital sodium, the mice were transcardially perfused with PBS (pH 7.0) containing 10 mmol/L glucose for 5 min to wash out blood components. Subsequently, the animals were perfused with 0.1 mg/ml FITC in PBS (pH 7.0) containing 10 mmol/L glucose for 5 min followed by PBS (pH 7.0) containing 5 U/ml heparin and 10 mmol/L glucose for 2 min, and then fixed by perfusion with 4% PFA (PH 8.0) for 10 min. The brains were removed and post-fixed in 4% PFA (PH 8.0) for 24 h at 4°C, followed by dehydration with 30% sucrose. Then, the brain tissues were cut into 20 µm thick coronal sections and mounted onto glass slides. After incubation with DAPI for 5 min, the sections were observed under a fluorescent microscope, and the number of perfused vessels in each slice was counted manually.

### Western Blot Analysis

The mice were euthanized under deep anesthesia using pentobarbital sodium (50 mg/kg, i.p.). The brain tissues in the ischemic area (about 50 mg) were homogenized in radioimmunoprecipitation assay (RIPA) buffer (Servicebio, China) containing 1% phenylmethanesulfonyl fluoride (PMSF). Protein concentration was determined using a bicinchoninic acid protein assay kit (Beyotime, Shanghai, China). Equal amounts of proteins were separated on 10% SDS-polyacrylamide gels, and then transferred onto nitrocellulose (NC) membranes (Millipore, United States). Next, the membranes were blocked with 5% skim milk for 1 h at room temperature, and then incubated with primary antibodies of rabbit anti-VEGFA (1:1000; Abcam, United Kingdom), rabbit anti-Ang-1 (1:10000; Abcam, United Kingdom), rabbit anti-Ang-2 (1:1000; Abcam, United Kingdom) or rabbit anti-β-actin (1:1000; Cell Signaling Technology, United States) at 4°C overnight. After washing three times with Tris buffered saline Tween (TBST), the membranes were incubated with secondary antibody of anti-rabbit IgG (H + L) (DyLight 800 Conjugate) (1:10000; Cell Signaling Technology, United States) for 1 h at room temperature. The protein bands were observed with Odyssey Infrared Imaging System (Li-COR Biosciences, Lincoln, NE, United States), and the intensity of each band was analyzed using Image J software.

### Reconstruction and Quantitative Analysis of 3D Cerebrovascular Network

#### Micro-Optical Sectioning Tomography

At 24 h after reperfusion, mouse brain samples were harvested and prepared according to a previously reported method (Wu et al., 2014; Li et al., 2010). After staining with the modified Nissl methods, the intact resin-embedded brain samples were sliced and imaged continuously using the BioMapping 1000 (OEBio Inc., Wuhan, China), which was based on the principle of MOST, with a sectioning thickness of 1 μm. Data collection was performed with an accuracy of 0.4 μm × 0.35 μm × 1 μm and the high-resolution 3D vascular network dataset of the whole brain was acquired (Wu et al., 2014; Wu et al., 2016). The total amount of uncompressed volume of each brain exceeded 2100 GB, covering more than 10,000 coronal sections. The raw images were degraded by uneven staining and illumination, and the artifacts were reduced using the oePreprocessing software (OEBio Inc., Wuhan, China). After the above preprocessing, the cross sections of cells and blood vessels could be clearly distinguished. The maximum intensity projection of the coronal sections showed the cytoarchitecture of vessels. Subsequently, for each whole brain dataset, three data blocks of the same size (600 μm × 600 μm × 600 μm) in each region (i.e., the cortex, striatum, and hippocampus) of ipsilateral hemisphere were randomly selected. The selected data blocks of each brain were also designated by the gray area in Supplementary Figure 2. Using the position of the data blocks in ipsilateral hemisphere as the reference, comparable data blocks of corresponding regions in contralateral hemisphere were then extracted. Initially, a skeleton of the blood vessels of each data block was developed using an automatic reconstruction method (Quan et al., 2016). Then tracking was refined manually to acquire the final vascular tracking results (Zhou et al., 2020). The tracked skeleton of blood vessels was then analyzed using the shape reconstruction algorithm to obtain the radius information of each skeletal point (Li et al., 2020). Blood vessels with diameters less than 6 μm were classified as microvessels (Wu et al., 2014). Then, the 3D data blocks were cut into several sub-blocks at equal intervals along the *z*-axis. The vascular tracking results in the sub-blocks contained the corresponding vascular skeleton and radius information, which were used for computing the vascular length density (VLD; total vascular length per tissue volume including large vessels and microvessels, m/mm^3^), and fractional vascular volume (FVV; total vascular volume relative to the total tissue volume, %). In addition, these data were further analyzed to obtain measurements for microvascular length density (MVLD) and fractional microvascular volume (FMVV; the definitions are the same as VLD and FVV, respectively, but only including microvessels) (Wu et al., 2014; Zhang et al., 2019).

#### Fluorescence Micro-Optical Sectioning Tomography

DyLight594-lycopersicon esculentum lectin (1 mg/ml, 0.2 ml per mouse; Vector Labs, United States) was administered *via* the tail vein 15 days after MCAO. The dye was allowed to circulate for 10 min. Mice were anesthetized and then transcardially perfused with normal saline and 4% PFA in turn. The brains were removed and placed in 4% PFA for 24 h. After embedding in resin (Gong et al., 2016; Gang et al., 2017), the whole brain specimens were imaged continuously using the fMOST system (BioMapping 5000; OEBio Inc., Wuhan, China). All the image tiles were obtained with a time-delay integration line scan charge-coupled device (Zhou YF. et al., 2019), and the images had a voxel size of 0.35 μm × 0.35 μm × 1 μm. Afterwards, the original images were preprocessed (Gong et al., 2016), and visualization and 3D data block contour rendering were performed using Amira software (version 5.2.2; FEI, France). The blood vessels of each data block (size of 400 μm × 400 μm × 400 μm) were tracked semi-automatically, and the VLD and FVV were calculated.

### Statistical Analysis

Statistical analyses were performed with GraphPad Prism software (version 9.0; La Jolla, CA, United States), and Shapiro-Wilk tests were used to test for normality. Data were represented as mean ± standard deviation (SD). For data exhibiting the normal distribution, unpaired t-tests were used for comparisons between two groups, and one-way analysis of variance (ANOVA) followed by Tukey’s post hoc tests were applied for comparisons among multiple groups. Otherwise, Mann–Whitney U tests were employed (the percentage of TUNEL^+^/NeuN^+^ cells). A *p*-value < 0.05 was considered statistically significant.

## Results

### Experiment I

The goal of Experiment I was to determine the optimum surgical parameters needed to create a TIA model for C57BL/6 mice that met the criteria of TIA. In general, the results of Experiment I presented below argued that an 8-min period of MCAO induced by the suture method is able to do this.

#### Cerebral Blood Flow Monitoring Revealed Ischemia and Reperfusion (Criterion 1).

**FIGURE 2:**
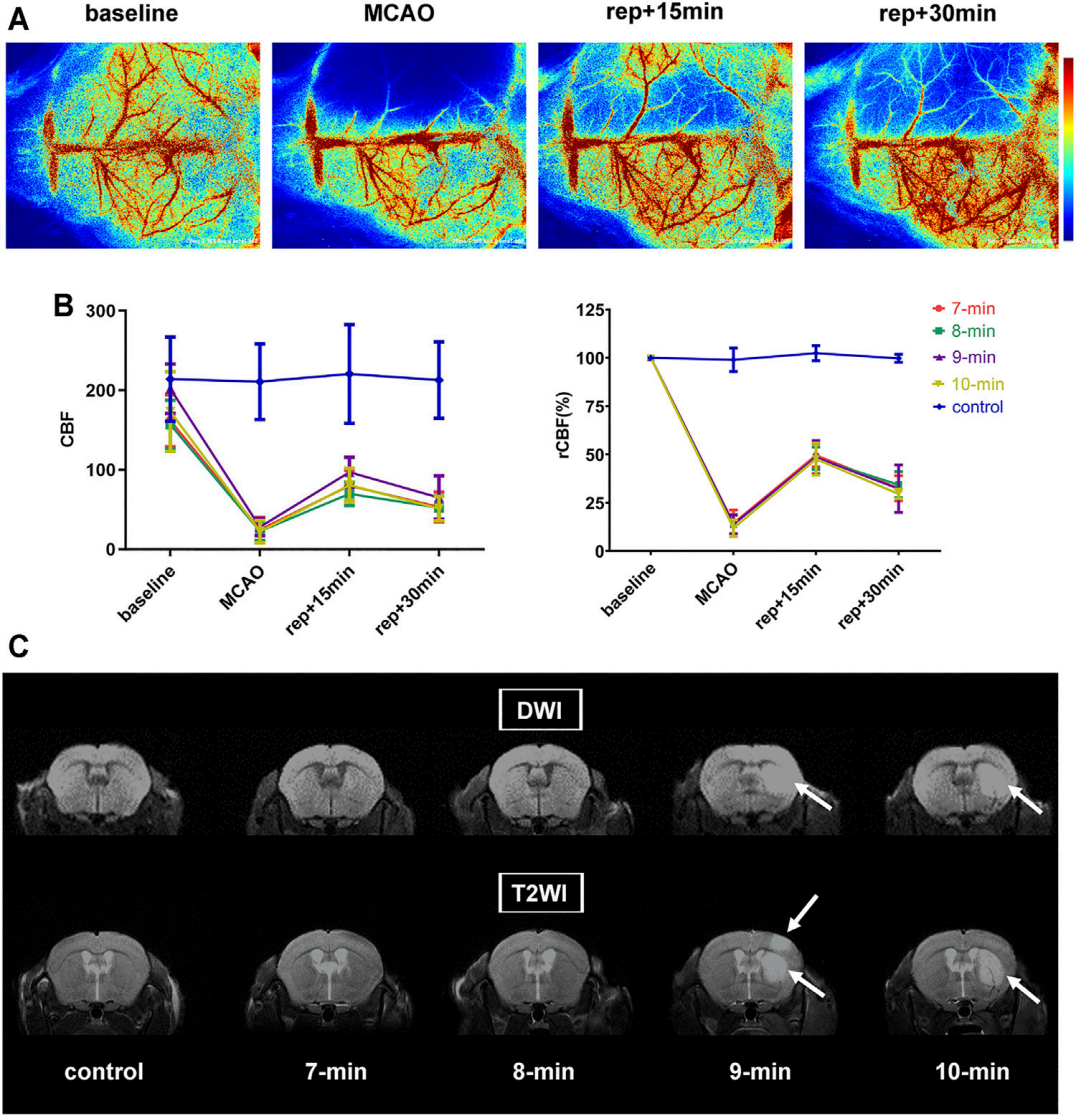
CBF measurements and MRI scans. **(A)** Representative laser speckle images showing CBF at pre-operation (baseline), occlusion (MCAO), and 15 and 30 min of reperfusion (rep+15 min and rep+30 min). **(B)** Broken line graph showing CBF and relative CBF (rCBF) of the ischemic hemisphere during operation (*n* = 6 per ischemia group, *n* = 3 in control group). **(C)** Representative diffusion weighted and T2-weighted images of each group (i.e., 7-, 8-, 9-, 10-min MCAO and control group) at 24 h post-reperfusion. The signal-enhancing regions in the right hemisphere (white arrows) indicated infarction. Data were presented as mean ± SD. rCBF, the percentage of CBF in the basal CBF.

LSCI was used to monitor CBF at 4 points (baseline, MCAO, 15- and 30-min of reperfusion) during surgery. Immediately after initiating MCAO, CBF values in the ischemic hemisphere decreased to 13.50 ± 5.09% of the baseline level, with no intergroup differences detected (*p* = 0.8586). After suture withdrawal, CBF values recovered significantly and reached 48.50 ± 6.99% of baseline at 15 min of reperfusion. In the subsequent reperfusion process, CBF values appeared to decrease, dropping to 32.11 ± 7.53% of baseline at 30 min of reperfusion. During reperfusion, no significant group differences in CBF values were detected (*p* = 0.9754 and 0.7971 for 15 and 30 min of reperfusion, respectively). CBF values of the control group displayed no obvious changes and remained at baseline levels throughout the experiment (*p* = 0.6187) (Figures 2A,B; Table 1).

**TABLE 1:** Physiological parameters and results of modeling.

	Groups (*n*)				
—	7-min (6)	8-min (6)	9-min (6)	10-min (6)	control (3)
Body weights (g) baseline^a^	22.7 ± 0.9	23.1 ± 0.9	22.9 ± 2.1	23.7 ± 1.6	23.9 ± 0.8
Reperfusion +24 h^b^	20.7 ± 0.9	21.4 ± 0.8	21.2 ± 1.6	21.8 ± 1.4	24.3 ± 0.8
rCBF (%) MCAO	14.40 ± 6.83	13.89 ± 5.11	13.80 ± 4.82	11.90 ± 4.32	99.00 ± 6.09
Reperfusion +15 min	49.57 ± 6.04	48.04 ± 5.83	48.58 ± 8.76	47.67 ± 8.32	102.43 ± 3.90
Reperfusion +30 min	32.53 ± 6.51	34.35 ± 6.81	32.31 ± 12.30	29.63 ± 2.45	99.71 ± 2.10
Neurological deficit (n, (*%*))	0 (0%)	0 (0%)	0 (0%)	0 (0%)	0 (0%)
MRI lesion (n, (*%*))	0 (0%)	0 (0%)	1 (16.67%)	2 (33.33%)	0 (0%)
Lesion volume in MRI (% of contralateral hemisphere)	0	0	8.06	5.35 ± 1.18	0

Grouping was based on the different durations of MCAO.

a Baseline body weights were similar across all groups (*p* = 0.6755).

b At 24 h of reperfusion, the average weight loss was 7.8% in MCAO mice (from 23.1 ± 1.4 to 21.3 ± 1.2 g; *p* < 0.0001), with no differences between the groups (*p* = 0.6816).

rCBF, relative cerebral blood flow, which is the percentage of CBF in the basal CBF.

#### Neurological Scores and Magnetic Resonance Imaging (Criteria 2 and 3).

No significant neurological deficits were detected in any group, but analysis of MRI data showed some detectable lesions (Figure 2C; Table 1). In the 10-min ischemia group, 2 of the 6 mice showed striatal infarcts on MRI (with lesion volumes of 6.18 and 4.51% of contralateral hemisphere), and the rest had no infarction. In the 9-min group, 1 of the 6 animals exhibited a striatal lesion with patchy cortical involvement (8.06% of the contralateral hemisphere). However, no evident abnormalities on MRI were found in any of the mice in the 8-min, 7-min and control groups. The animals subjected to 8-min MCAO also presented no infarcts 7 days after reperfusion, indicating that no delayed ischemic lesions occurred.

Taken together, neither neurological deficits nor cerebral infarcts occurred in C57BL/6 mice when the MCA was blocked for no more than 8 min. Therefore, a TIA model can be achieved in this strain by intraluminal filament occlusion of the MCA for 8 min or shorter.

### Experiment II

#### Cerebral Blood Flow Monitoring by Laser Speckle Contrast Imaging or Laser Doppler Flowmeter Showed a Consistent Trend in Transient Ischemic attack Mice

**FIGURE 3:**
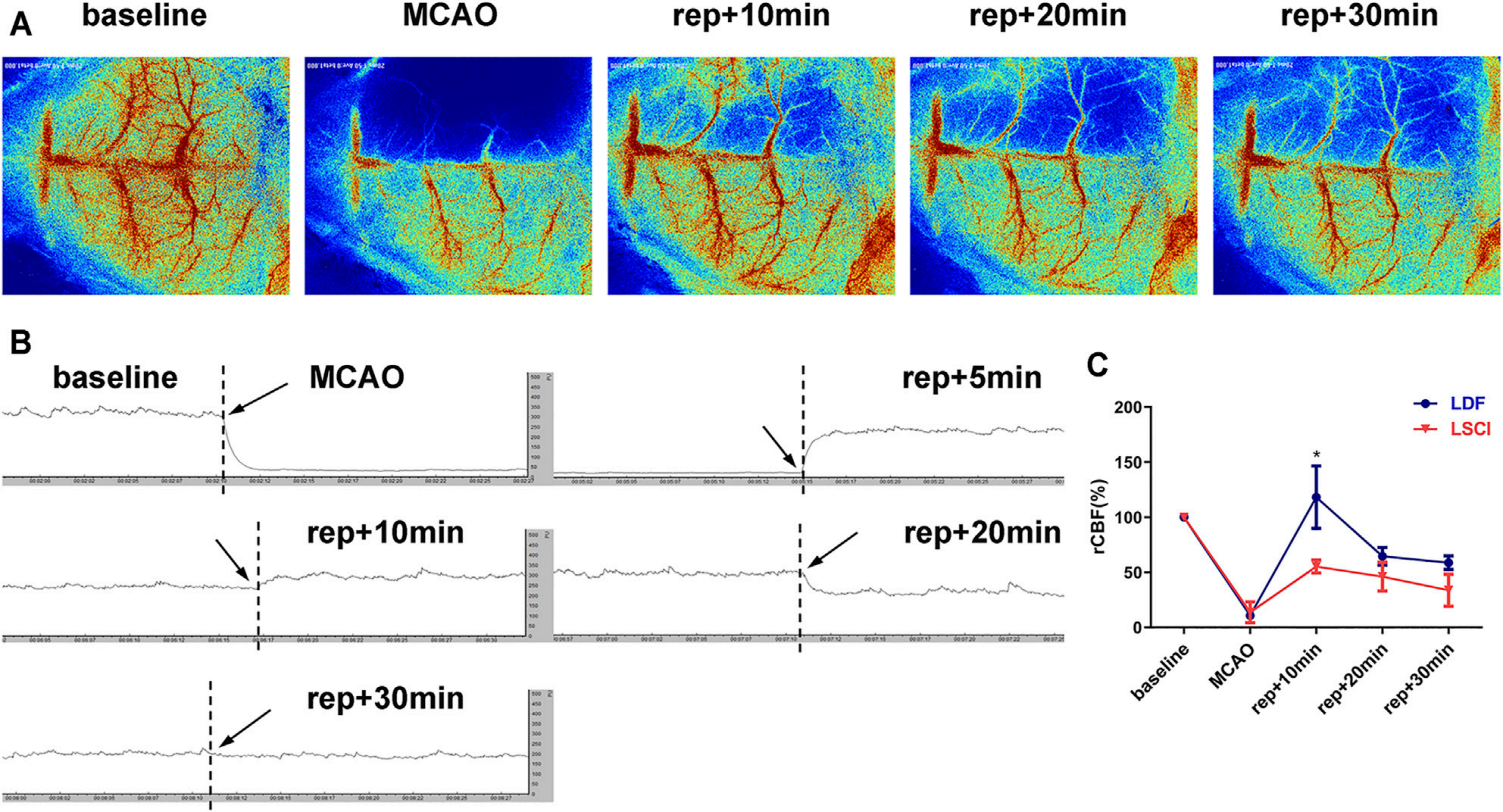
CBF monitoring by laser speckle contrast imaging (LSCI) or laser Doppler flowmeter (LDF)showed a consistent trend in TIA mice. **(A)** Representative laser speckle images showing CBF at pre-operation (baseline), occlusion (MCAO), and 10, 20 and 30 min of reperfusion (rep+10 min, rep+20 min and rep+30 min) of 1 mouse subjected to 8-min ischemia. **(B)** Representative laser Doppler images of the ischemic hemisphere. **(C)** Broken line graph showing relative CBF of the ischemic hemisphere during operation in the LDF- and LSCI-monitored groups (*n* = 3 per group). Data were presented as mean ± SD and determined by unpaired *t*-test. P ∗  < 0.05 vs. LSCI-monitored group.

To compare the effectiveness of the two methods commonly used to measure CBF during surgery, CBF was monitored with either LSCI or LDF during the establishment of the above-mentioned TIA model (the mice subjected to 8-min MCAO). As shown in Figure 3, MCAO caused an immediate decrease in CBF of the ischemic hemisphere, and the values measured by LSCI or LDF were 13.73 ± 9.48% or 10.80 ± 0.49% of the baseline level, respectively, with no intergroup differences (*p* = 0.7000). After suture withdrawal, CBF values recovered significantly, and reached 55.19 ± 5.83% (LSCI-monitored group) or 118.19 ± 28.33% (LDF-monitored group) of baseline at 10 min of reperfusion. Thereafter, CBF values appeared to drop in the both groups. At 20 min of reperfusion, the values decreased to 46.08 ± 12.92% (LSCI group) or 64.58 ± 8.04% (LDF group) of baseline, and at 30 min, 33.82 ± 14.52% or 58.78 ± 6.21%, respectively. Throughout reperfusion, the recovery of CBF values in the LDF-monitored group was more obvious compared to LSCI-monitored group, but the only statistical difference between the two groups existed at 10 min post-reperfusion (*p* = 0.0196 for 10 min of reperfusion; *p* = 0.1031 and 0.0521 for 20 and 30 min).

### Experiment III

#### TUNEL and NeuN Fluorescent Double-Label Staining Indicated Neuronal Apoptosis After Transient Ischemic attack

**FIGURE 4:**
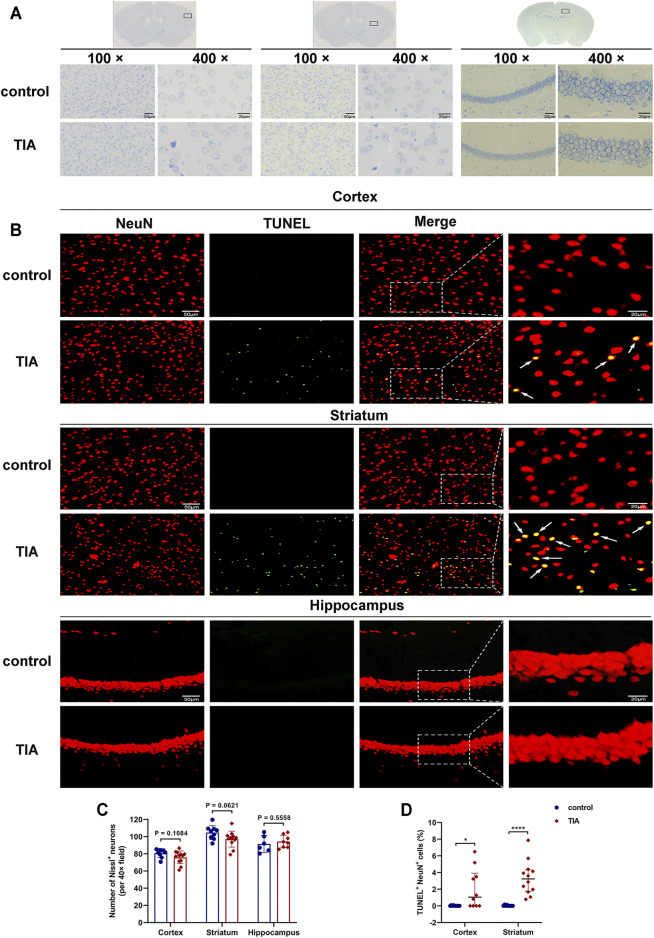
TUNEL and NeuN fluorescent double-label staining indicated neuronal apoptosis after TIA. **(A)** Nissl-stained images of the cortex, striatum and hippocampus (100×, scale bar = 50 μm; 400×, scale bar = 20 μm). **(B)** Representative TUNEL staining for neuron (red) and TUNEL (green) in cortex, striatum and hippocampus acquired from the ischemic hemisphere of control and TIA mice. Low-magnification, full field (scale bar = 50 μm) and high-magnification, partial visual fields (indicated by the white frame; scale bar = 20 μm) were displayed. Arrow points represented apoptotic neurons. **(C)** Bar graphs showing the number of intact neurons in the ipsilateral cortex, striatum and hippocampus of the control and TIA groups (*n* = 3 and 4, respectively). Data were presented as mean ± SD and determined by unpaired *t*-test. **(D)** Dot plots showing the percentage of TUNEL and NeuN double-positive cells in the ischemic hemisphere. Data were presented as median and interquartile range and determined by Mann-Whitney *U* test. P ∗  < 0.05; P ∗∗∗∗  < 0.0001 control group vs. TIA group.

Nissl staining showed that, 24 h after surgery, the neurons in the cortex and striatum of the ischemic hemisphere of the TIA mice were clear in shape and intact in structure, except for a few scattered neurons exhibiting shrunken and deeply stained (Figure 4A). The quantitative results (Figure 4C) indicated that the number of intact neurons in these two brain regions in the TIA group had a trend towards decrease compared with the control group (75.89 ± 7.64 vs. 80.70 ± 4.82 in cortex; 97.08 ± 9.22 vs. 104.74 ± 8.09 in striatum; *p* > 0.05, TIA group vs. control group). Whereas in the hippocampus, there were no neuronal defects in TIA mice compared to the control group (94.25 ± 7.33 vs. 91.50 ± 9.70; *p* = 0.5558), and the neurons were tightly arranged, with no obvious pathological changes.

To evaluate neuronal apoptosis after TIA, TUNEL/NeuN double immunostaining was performed. The results indicated that the number of TUNEL/NeuN double-positive cells increased in the ipsilateral cortical and striatal regions of the TIA group compared to those of the control group (*p* < 0.05; Figures 4B,D). However, no significant TUNEL/NeuN double-positive cells were observed in the hippocampal region of the TIA and control mice. Together, these results suggest that 8-min MCAO induced neuronal apoptosis in the cortex and striatum of TIA mice.

#### Cerebral Vasculature Abnormalities in Transient Ischemic attack Mice

**FIGURE 5:**
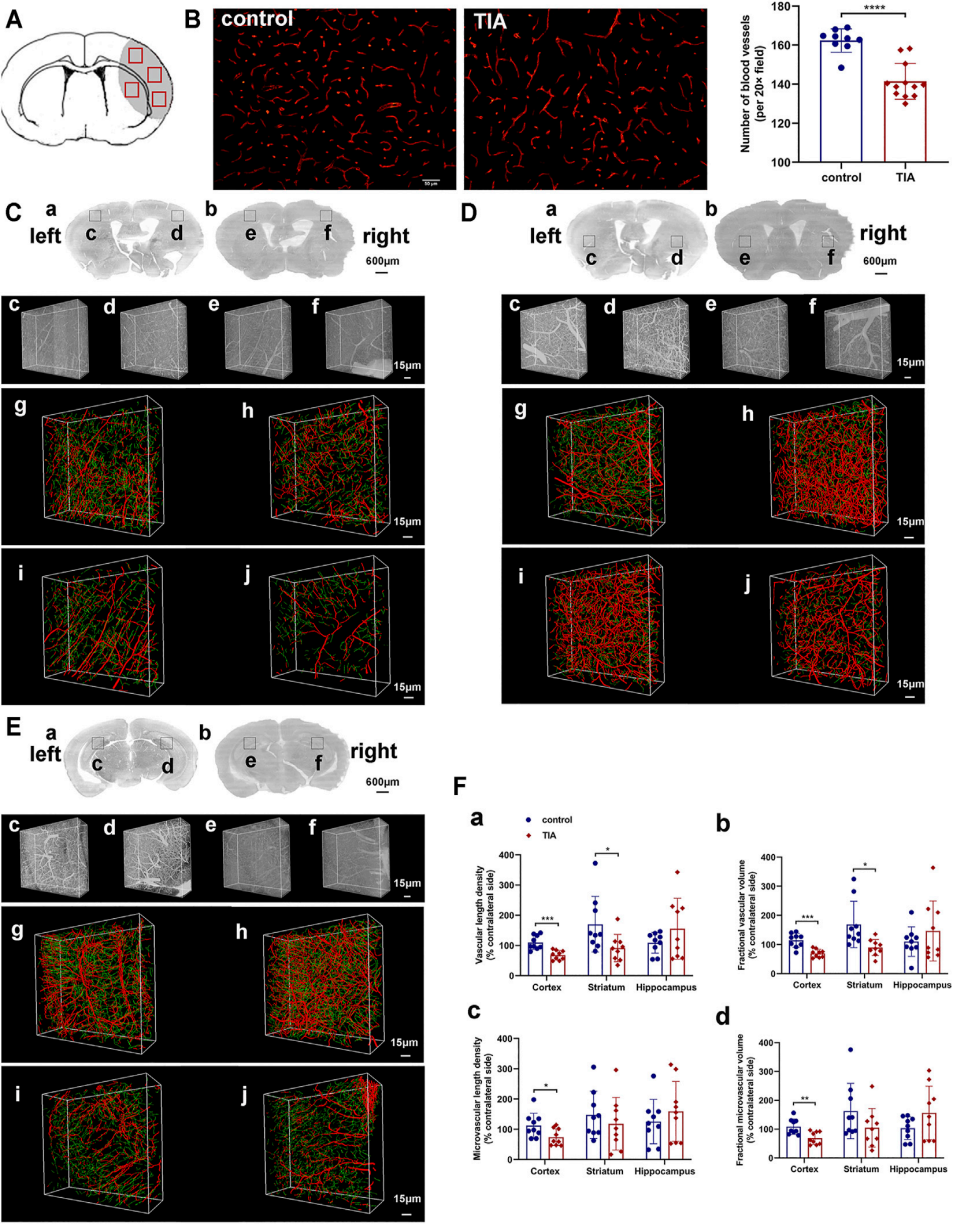
Cerebral vasculature abnormalities in TIA mice. **(A)** A schematic diagram of the immunofluorescence images. The gray area represents the ischemic brain regions during MCAO surgery, and the red boxes represent the regions selected for imaging in each section. **(B)** Representative image showing immunofluorescent staining for CD31 in the ischemic hemisphere of control and TIA mice (scale bar = 50 μm). Bar graphs showing the quantification of the CD31^+^ microvessels (*n* = 3 in control group, *n* = 4 in TIA group). Representative 3D reconstruction images of cerebrovascular network in the cortex **(C)**, striatum **(D)** and hippocampus **(E)** at 24 h post-MCAO. **(C) (a)**, **(D) (a)** and **(E) (a)** Coronal brain projection images of different layers in the control group. **(C) (b)**, **(D) (b)** and **(E) (b)** Coronal brain projection images in the TIA group from the same layers as **(C) (a)**, **(D) (a)** and **(E) (a)**, respectively. In **(C–E)**, **(c–f)** are the raw images of vessel structures in the boxed areas of **(a)** and **(b)**. In **(C–E)**, **(g–j)** are the 3D reconstruction cerebrovascular images of **(c–f)**, respectively. **(g)** and **(h)** acquired from the control group; **(i)** and **(j)** acquired from the TIA group. The images showed the ischemic areas **(h,j)** and the contralateral areas **(g,i)**. The red labeling shown in the images represented large vessels (diameter >6 μm), and the green labeling represented microvessels (diameter <6 μm). **(F)** Statistical analysis of the vascular length density **(a)**, fractional vascular volume **(b)**, microvascular length density **(c)** and fractional microvascular volume **(d)** of the ischemic hemisphere. The data was expressed as [(the ischemic hemisphere value/the contralateral hemisphere value) × 100%] (*n* = 3 mice per group; *n* = 3 data blocks per brain region, i.e., cortex, striatum and hippocampus). Data were presented as mean ± SD and determined by unpaired *t*-test. P  ∗  < 0.05; P ∗∗  < 0.01; P ∗∗∗  < 0.001; P ∗∗∗∗  < 0.0001 control group vs. TIA group.

Immunofluorescent staining showed that the number of CD31^+^ microvessels decreased in the ischemic hemisphere of TIA mice compared to the control group at 24 h after MCAO (*p* < 0.0001; Figure 5B). To ensure complete and comprehensive observation of the cerebral vasculature changes after TIA, we used MOST to reconstruct the blood vessels of the ischemic and contralateral hemisphere, and examined the 3D density of the vessels at submicron resolution. The images of MOST revealed that the vascular density was lower in the ipsilateral cortical, striatal and hippocampal regions of TIA mice, as compared with the control group (Figures 5C–E). The quantitative data on the vasculature of the three brain regions (Figure 5F, Supplementary Table 2) showed that the VLD, FVV, MVLD and FMVV were remarkably reduced in the ipsilateral cortex of TIA mice compared to the control group (*p* < 0.05). Similarly, in the ipsilateral striatum, the VLD and FVV of TIA mice exhibited a significant decrease relative to the control group (*p* < 0.05), while a decreasing trend in the MVLD and FMVV was noted (*p* > 0.05). However, no significant difference between the groups was detected in the ipsilateral hippocampal vasculature (*p* > 0.05). Overall, the results indicated different abnormalities of the vasculature in various brain regions of our TIA mice, with the cortical and striatum regions displaying decreased vascular density.

### Experiment IV

#### Dl-3-N-Butylphthalide Promoted Angiogenesis After Transient Ischemic attack by Increasing the Expression of Angiogenic Growth Factor

**FIGURE 6:**
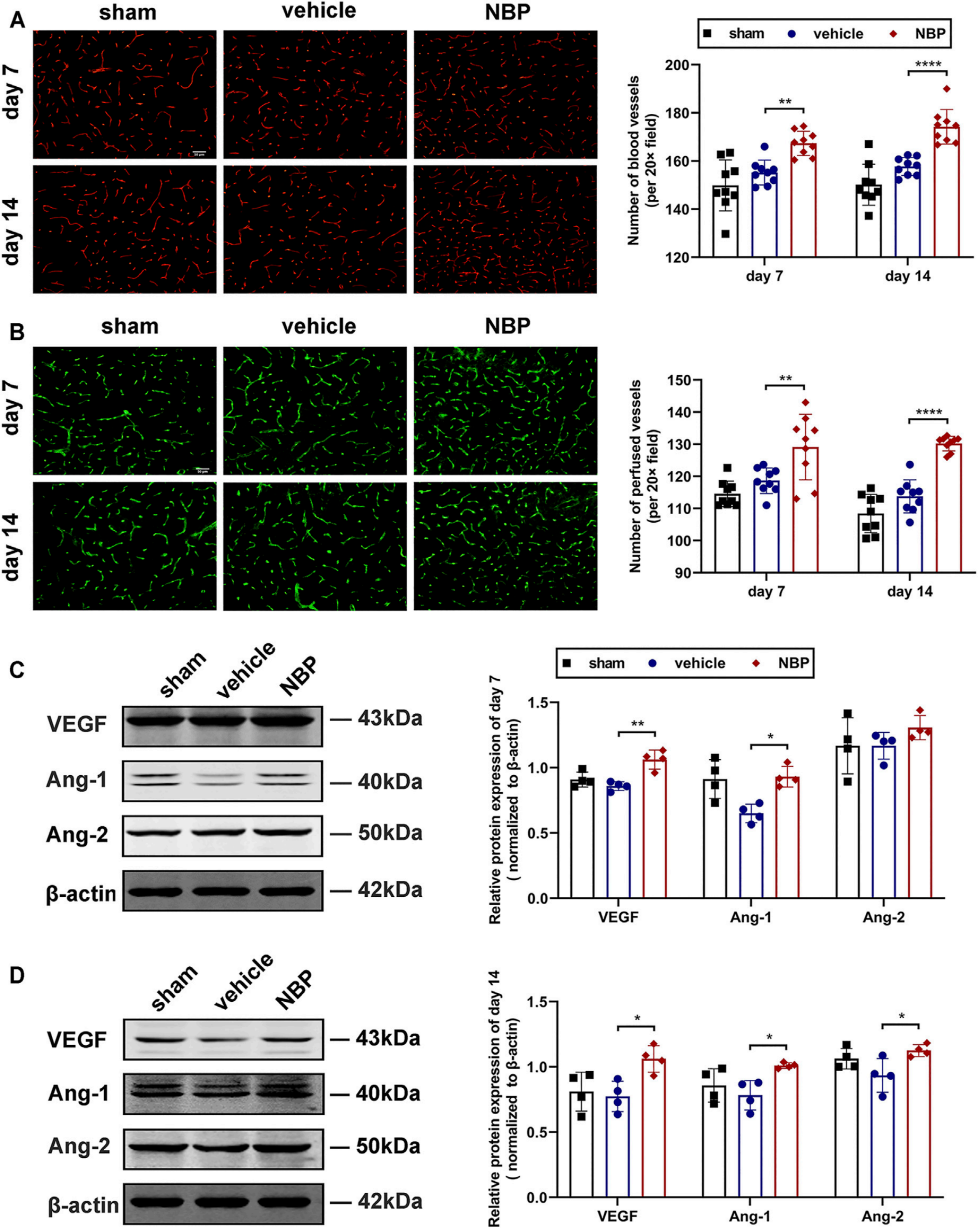
NBP promoted angiogenesis after TIA by increasing the expression of angiogenic growth factor. **(A)** Representative images showing the immunofluorescent staining for CD31 in the ischemic hemisphere at 7 and 14 days post-MCAO (scale bar = 50 μm; the regions selected for imaging were the same as Figure 5A). Bar graphs showing the quantification of the CD31^+^ microvessels (*n* = 3 per group). **(B)** Representative images showing the FITC-labeled perfused microvessels in the ischemic hemisphere (scale bar = 50 μm). Bar graphs showing the vascular quantification (*n* = 3 per group). Representative images of western blot analysis for VEGF/Ang-1/Ang-2 in the ischemic hemisphere at 7 **(C)** and 14 **(D)** days after MCAO. Bar graphs showing the data quantification (*n* = 4 per group). Data were presented as mean ± SD and determined by one-way ANOVA with Tukey’s post-hoc test.  P  ∗ <0.05; P ∗∗ < 0.01; P ∗∗∗∗ < 0.0001 vehicle-treated group vs. NBP-treated group.

In Experiments I-III, we developed a TIA model in C57BL/6 mice, and detected a decreased vascular density in the ischemic hemisphere of TIA mice. To explore the effects of NBP on angiogenesis after TIA, we used immunofluorescent staining for CD31 to examine vascular density 7 and 14 days after MCAO. The results showed that the number of CD31^+^ microvessels in the ischemic hemisphere of the NBP-treated group was significantly increased at 7 and 14 days post-MCAO compared to those of the vehicle-treated group (*p* < 0.01; Figure 6A), indicating that NBP promoted angiogenesis in TIA mice. The FITC method was used to determine if these microvessels were functional. We found that the number of perfused microvessels in the ischemic hemisphere of the NBP-treated group was also significantly higher than that of the vehicle-treated group at 7 and 14 days post-MCAO (*p* < 0.01; Figure 6B).

To further clarify the factors promoting the increase in microvessels, the expression of angiogenic growth factor was detected by western blot analysis. Our results showed that at 7 days post-MCAO, the protein expression of VEGF and Ang-1 were significantly increased in the NBP-treated group compared to the vehicle-treated group (*p* < 0.05; Figure 6C). At 14 days post-MCAO, the protein levels of VEGF, Ang-1 and Ang-2 were significantly up-regulated in the NBP-treated group compared with the vehicle-treated group (*p* < 0.05; Figure 6D).

**FIGURE 7:**
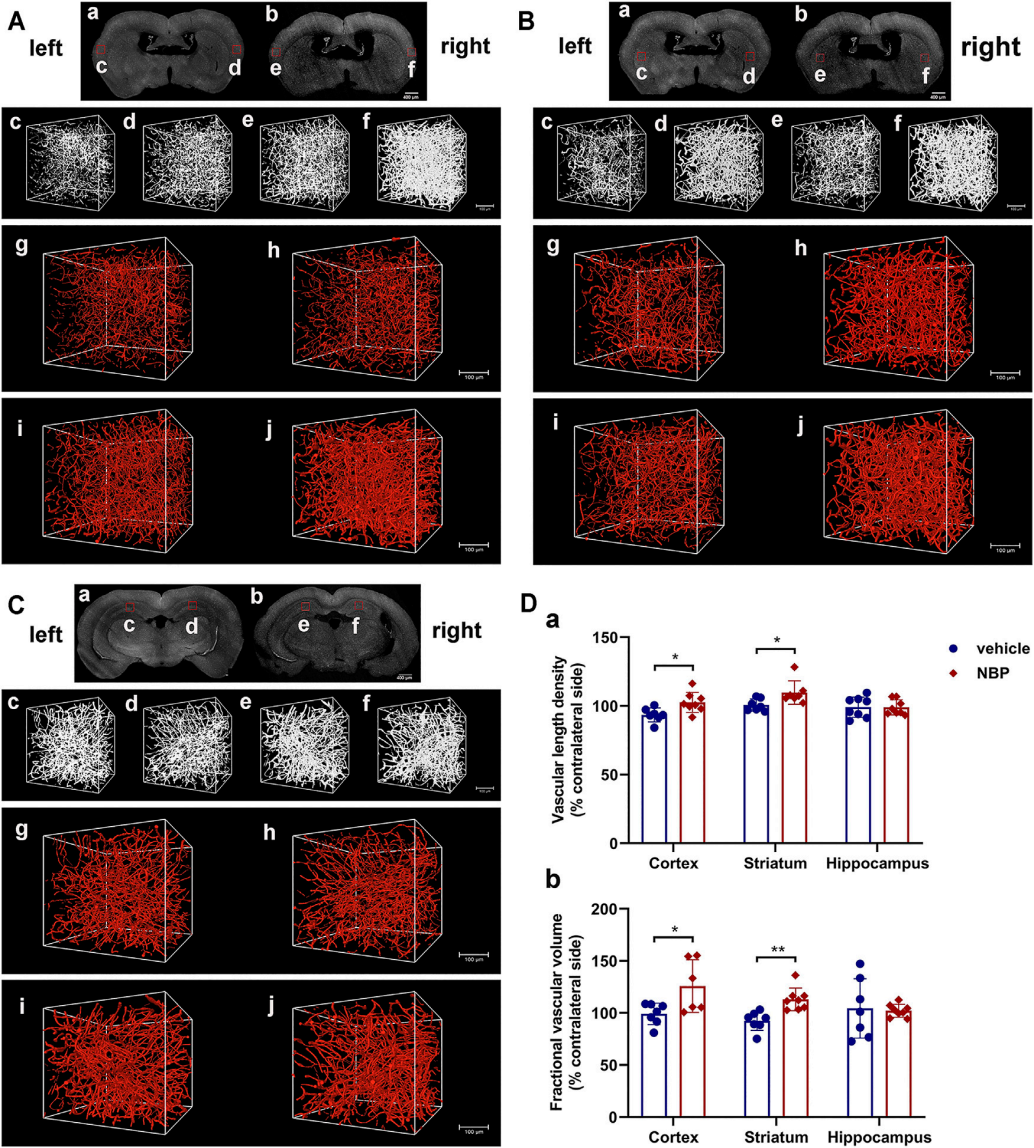
NBP promoted the vascular density and improved the vascular network. Representative reconstructed images of cerebrovascular network by fMOST in the cortex **(A)**, striatum **(B)** and hippocampus **(C)** at 15 days after MCAO. **(A) (a)**, **(B) (a)** and **(C) (a)** Coronal brain projection images in the vehicle-treated group. **(A) (b)**, (**B) (b)** and (**C) (b)** Coronal brain projection images in the NBP-treated group from the same layers as **(A) (a)**, **(B) (a)** and **(C) (a)**, respectively. In **(A–C)**, **(c–f)** are the original images of vessel structures in the boxed areas of **(a)** and **(b)**. In (**A–C)**, **(g–j)** are the 3D reconstructed cerebrovascular images of **(c–f)**, respectively. **(g)** and **(h)** acquired from the vehicle-treated group; **(i)** and **(j)** acquired from the NBP-treated group. The images showed the ischemic areas **(h,j)** and the contralateral areas **(g,i)**. **(D)** Statistical analysis of the vascular length density **(a)** and fractional vascular volume **(b)** of the ischemic hemisphere (*n* = 3 per group). Data were presented as mean ± SD and determined by unpaired *t*-test. P  ∗  < 0.05; **P < 0.01 vehicle-treated group vs. NBP-treated group.

Next, we employed fMOST to fully investigate the changes in the 3D density of the vessels after NBP intervention, which could present all the details of the vascular pattern. As shown in Figures 7A,B, the reconstructed images of cerebral vascular network revealed that the administration of NBP increased the vascular density in the cortex and striatum of the ischemic hemisphere, and the VLD and FVV in these two regions in the NBP-treated group were significantly higher than those in the vehicle-treated group (*p* < 0.05; Figure 7D). Nevertheless, there were no significant differences in the VLD and FVV of the ipsilateral hippocampus between the NBP group and the vehicle group (*p* > 0.05; Figures 7C,D). These results further confirmed that NBP promoted the vascular density, and improved the vascular network in the lesion area after TIA.

## Discussion

Because of the absence of residual neurological deficits and imaging-based evidence of brain infarction, TIA was previously considered a benign event. However, there is growing evidence that TIA is a clinical entity that heralds imminent ischemic stroke (Hill et al., 2004; Rothwell and Warlow, 2005; Giles and Rothwell, 2007; Johnston et al., 2007) and is associated with increased incidence of depression and cognitive impairment (El et al., 2012; Moran et al., 2014; van Rooij et al., 2014; Turner et al., 2016). Even so, research on TIA is relatively rare. One reason for this is the lack of an appropriate and available animal model, making it difficult to further explore the pathology, mechanisms and potential targeted treatments of TIA. In this study, we utilized the suture MCAO method and followed the three established criteria of TIA models (Pedrono et al., 2010; Durukan et al., 2017) to evaluate the outcomes of 7–10 min of ischemia in C57BL/6 mice to establish an optimal TIA model. The criteria are as follows: 1) objective evidence of ischemia and reperfusion, primarily relying on CBF monitoring to confirm adequate occlusion and reperfusion; 2) no permanent neurological deficit; and 3) no acute cerebral infarction. The importance of the second and third criteria is to make the animal model achieve a clinical relevance *via* neurological testing and MRI examination at 24 h of reperfusion. Our results showed that these criteria were met for MCAO of 8 min or shorter in C57BL/6 mice, indicating the successful establishment of the TIA model, whereas MCAO that exceeded 8 min induced ischemic lesions that are detectable by MRI and was equivalent to cerebral infarction. Thus, the threshold point separating TIA and cerebral infarction in C57BL/6 mice is between 8 and 9 min MCAO.

Durukan (Durukan et al., 2017) and Pedrono (Pedrono et al., 2010) also established TIA models in Wistar rats and NMRI mice, respectively, by using the suture MCAO method. Their results indicated that neither neurological deficits nor cerebral infarcts occurred when the MCA was occluded for no more than 10 min, which was regarded as a cut off duration of the TIA model. However, in our study, a 10-min period of MCAO was enough to induce cerebral infarction in C57BL/6 mice. Moreover, 10 min of ischemia could cause MRI lesions in SD rats (Fan et al., 2017). ICR-CD1 mice also exhibited striatal infarcts on MRI after 10-min ischemia (Berthet et al., 2011). These studies show that different species present various outcomes after experiencing a consistent duration of MCAO, proving the tolerance of different species to cerebral ischemia is diverse. The mechanism for this variation is still unclear, and remains to be investigated in future research. The inherent properties of the species, including differences in the composition of the circle of Willis (cerebrovascular anatomy), the formation of collateral circulation, and the distribution of gray and white matter may play important roles (Durukan et al., 2017). Barone et al. (1993) reported obvious differences between BDF, CFW, and BALB/C mice in sensitivity to cerebral ischemia that were partly related to the variation in the posterior communicating arteries among the strains. Connolly et al. (1996) also compared the outcomes of permanent focal cerebral ischemia in three commonly used mouse strains (CD-1, C57BL/6 and 129/J) and found that the infarct volume and neurological scores were significantly distinct between the three strains. However, in their study, strain differences in the composition of the circle of Willis were not apparent, suggesting strain differences in ischemic effects might result from other non-anatomical factors. Notably, even within the same strain, sensitivity to ischemia can be affected by differences in origin or vendors, which may be ascribed to genetic divergence caused by different start-up stocks and breeding strategies (Oliff et al., 1995). In addition, factors such as the animal’s body weight, the filament diameter, anesthesia, temperature, and even the surgery skills of the experimenter play roles on the outcome of the model. Therefore, preliminary experiments are required to determine their specific infarct thresholds when our TIA model is applied to other strains and species.

The MCA is the most frequently affected cerebral vessel in human ischemic cerebral vascular disease. In this study, the TIA model was developed on the basis of the suture MCAO method, with a suture blocking the origin of the MCA that causes a sharp decrease in the blood flow to the frontoparietal cortex and striatum. The MCA belongs to the internal carotid artery system (i.e., anterior circulation), and thus, the MCAO model will cause anterior circulation ischemia, and our study also focused on the MCA blood supply area. While the posterior circulation brain regions are less likely to be damaged in the transient MCAO model (especially our TIA model induced by 8-min MCAO), because their blood supply are derived from the vertebrobasilar system (including the vertebral artery, basilar artery and posterior cerebral artery). So far, fewer animal models have been developed for posterior circulation ischemia (PCI). Some methods similar to anterior circulation ischemia, including vertebrobasilar vessels clipping, ligation, photochemical and embolization, have been used to induce PCI in rats (Henninger et al., 2006), gerbils (Hata et al., 1993), cats (Nakahara et al., 1991) and dogs (Gnyawali et al., 2020). However, there is still a lack of mouse models of PCI, which may be due to the fact that PCI affects the brain stem (the vital center), the mice are prone to death and be short-lived. Moreover, it is difficult to separate the vertebral artery and basilar artery in mice, and so the success rate of the model is low. Therefore, the methods for establishing a vertebrobasilar (posterior circulation) TIA model in mice remain to be further explored in the future.

LSCI is a non-contact, minimally-invasive and full-field CBF monitoring method, with the ability to provide real-time two-dimensional CBF imaging with high temporal and spatial resolution, which is obviously superior to laser Doppler scanning technology (Ayata et al., 2004; Wang et al., 2020). In this study, LSCI was adopted to monitor the CBF changes during the establishment of the model. The results showed that decline in CBF during MCAO was basically consistent with that reported in the literature (Durukan et al., 2017; Pedrono et al., 2010), whereas the degree of CBF recovery after suture withdrawal was lower, returning to about 48% of the baseline values at 15 min of reperfusion followed by approximately 32% at 30 min. We found that CBF did not recover gradually over time during reperfusion, but instead decreased between 15 and 30 min of reperfusion. Thus, we conducted a separate experiment to compare LSCI and LDF monitoring of CBF (the most commonly methods for CBF monitoring) during TIA surgery. Both methods found a consistent trend of gradually decreasing CBF from 10 to 30 min of reperfusion. Furthermore, we observed that the CBF values in the LSCI-monitored group recovered to 55% of the baseline level at 10 min of reperfusion, but the subsequent measurements at 20 and 30 min of reperfusion did not reach 50% of the baseline. In contrast, the CBF values in the LDF-monitored group all exceeded 50% of baseline throughout the reperfusion. Moreover, at every time point of reperfusion, the recovery of CBF in the LDF-monitored group was more obvious than that in the LSCI group. This may be because LDF provided a single-point measurement of CBF within 1 mm^3^, whereas LSCI measured the average CBF within a circular ROI with a diameter of 2.5 mm. Previous studies also reported (Schmid-Elsaesser et al., 1998; Chen et al., 2015) that after filament withdrawal, animals would first undergo a rapid hyperemia process, known as the post-ischemic reactive hyperperfusion period, during which CBF exceeded baseline levels. After that, a gradual decrease in CBF was observed, called the delayed post-ischemic hypoperfusion state, which persisted until the end of the recording period at 30 min of reperfusion, and the CBF could not reach the normal baseline values. In Experiment I, the CBF values in each group during reperfusion did not reach the minimum standard in the literature (50% of baseline values) (Durukan et al., 2017), which might be ascribed to differences in the experimental conditions, the CBF measurement equipment, the selected measured location and the size of ROI. Additionally, we selected only two time points of reperfusion (i.e., 15 and 30 min) for CBF measurement, and in Experiment II, we observed that CBF decreased between 10 and 30 min of reperfusion, thus the time point at which CBF returned to 50% of the baseline might not have been recorded. Future studies should increase the time points for CBF measurement during reperfusion (e.g., 1, 5, 10, 15, 20 and 30 min) and continue to monitor CBF after 30 min of reperfusion. If conditions permit, CBF can even be monitored throughout the reperfusion to determine the CBF changes during the establishment of TIA model.

Neurological testing and MRI examinations were performed at 24 h after reperfusion because by this time, cerebral infarcts were proven to be complete (Pedrono et al., 2010). Interestingly, the occurrence of infarction was not always accompanied by neurological deficits. In our 9- and 10-min MCAO groups, although cerebral infarcts were shown on MRI, the neurological scores were normal (neurological function inconsistent with imaging), possibly because this neurological score was sensitive only to general sensorimotor deficits. Tang et al. (2014) found that no infarcts were detected with TTC staining in C57BL/6 mice subjected to 10-min ischemia, whereas in our study, a 10-min period of MCAO induced lesions in this strain that were detectable by MRI. This is because TTC staining mainly relies on an enzymatic reaction and cannot exclude small-scale infarcts, and thus is not as accurate as MRI.

Generally speaking, there is no cerebral infarction detectable in MRI images of TIA patients. However, a negative MRI result does not mean that the brain tissue is uninjured. Pedrono et al. (2010) found that in NMRI mice, MCAO as brief as 2.5 min caused slight histopathological changes, and their TIA mice with 10-min ischemia presented selective neuronal necrosis and apoptosis. Similarly, there were scattered ischemic neuronal changes and a small amount of neuronal apoptosis in the TIA model of Wistar rats (10-min MCAO), which did not worsen from 24 h to 7 days after reperfusion (Durukan et al., 2017). Ejaz et al. (2015) utilized 15-min distal MCAO to establish a TIA model in spontaneously hypertensive rats, and their histopathological examination revealed selective neuronal loss (SNL) and microglial activation in the cortex. In C57BL/6 mice, one or three 5-min MCAO with 10-min reperfusion intervals produced no infarcts detectable with TTC staining, but induced scattered neuronal damage in 20% of the mice (Stagliano et al., 1999). Additionally, studies reported that SNL occurred in the striatum or cortex after a short period of ischemia, which was a common phenomenon following 5- to 20-min MCAO in the rodent (Baron et al., 2014). In Wistar rats, the severity of SNL increased with the longer occlusion duration, from 5 to 20% between 10- and 20-min MCAO (Garcia et al., 1997). Our results showed that TIA model with C57BL/6 mice experiencing 8-min MCAO exhibited evidence of neuronal apoptosis in the cortex and striatum to a statistically greater extent than seen in the control group, together with the number of Nissl-stained neurons in these two regions reducing by 5.97 and 7.31%, respectively, compared with the control group. It is worth mentioning that the severity of apoptosis also increases with increased ischemia duration (Durukan et al., 2017), thus the proportion of TUNEL/NeuN double-positive cells after TIA is significantly lower than that of cerebral infarction (Yu et al., 2016; Zhao et al., 2019).

It has not been clear from previous research whether there are abnormalities in cerebral vasculature after TIA. However, several studies found that although the neurological functions of TIA patients appear to completely recover, hypoperfusion on the ischemic hemisphere is detectable with CT perfusion imaging (CTP), arterial spin label imaging (ASL) and other imaging techniques (Prabhakaran et al., 2011; Zaharchuk et al., 2012; Qiao et al., 2013), suggesting that TIA may have impacts on the cerebral vasculature. MOST is a new method of brain imaging that can provide fine-structure tomography of a centimeter-size sample at submicron resolution, and clearly distinguish the morphology and spatial locations of the neurovascular unit (Li et al., 2010; Wu et al., 2016). By combining modified Nissl staining with MOST, the vascular structure of the entire mouse brain can be directly visualized, and vascular abnormalities below capillaries can be identified. In this experiment, immunofluorescent staining results showed that the number of CD31^+^ microvessels of TIA mice was lower than that of the control group. Subsequently, the advanced MOST technology was used to perform 3D high-resolution visual reconstruction and quantitative analysis of the mouse cerebrovascular system to further investigate the vasculature changes after TIA. We found different abnormalities of the vasculature in various brain regions of TIA mice. In the cortical and striatal regions, the VLD and FVV in the ischemic hemisphere of TIA mice were significantly lower than that of the control group, and in the ipsilateral cortical region, the MVLD and FMVV of TIA mice were also remarkably decreased. This may account for the reported hypoperfusion phenomenon after TIA, and the long-lasting hypoperfusion may be one of the causes of cognitive impairment. Nevertheless, in the hippocampal region, no significant difference was detected between the two groups, which might be related to the surgical model we selected. That is, the suture MCAO model mainly results in damage to the frontal and parietal cortex and striatum, and generally does not involve the hippocampus.

NBP is a clinical drug used to treat ischemic stroke in China. NBP can act on multiple links of cerebral ischemia pathology, and play a protective role on cerebral infarction through anti-inflammatory, anti-apoptosis, antioxidation and anti-thrombosis mechanisms (Chang and Wang, 2003; Qin et al., 2019; Yang et al., 2019). Angiogenesis is extremely important in the recovery of neurological function after cerebral ischemia. Previous reports found that NBP could also promote angiogenesis after cerebral infarction by up-regulating the expression of angiogenic growth factors, and thereby reduce ischemic brain injury (Liu et al., 2007; Liao et al., 2009; Zhou YF. et al., 2019). However, most of the research on NBP involved ischemic stroke, and its effects on TIA remain unclear. A recent study showed that NBP injection could prevent stroke following TIA (Zhang et al., 2020). Therefore, based on the detection of cerebral vasculature abnormalities in TIA mice, we further explored the therapeutic effects of NBP on TIA. These results indicated that NBP significantly increased the number of the CD31^+^ microvessels. More importantly, the number of perfused microvessels also increased, indicating that these were also functional. Additionally, the expression of angiogenic growth factors including VEGF, Ang-1 and Ang-2 in the NBP-treated group also increased. To achieve a more complete visualization of angiogenesis after TIA, we employed fMOST to reconstruct the entire cerebrovascular network of the mice in the vehicle- and NBP-treated groups 15 days after MCAO. The results indicated that the VLD and FVV of the ipsilateral cortex and striatum were significantly increased in the mice that were treated with NBP. Unlike MOST, which is based on traditional staining methods (e.g., Nissl staining) and imaging under a bright field microscope, fMOST utilizes fluorescent confocal microscope to perform high-resolution imaging of fluorescence-labeled samples. In this study, we used intravenous injection of fluorescent dye to label cerebral vessels combined with fMOST technology. However, after imaging, most of the blood vessels observed were microvessels, and almost no large vessels were found in the data blocks included in the analysis. This limitation might be related to the staining characteristics of lycopersicon esculentum lectin, which primarily labels the endothelium of small vessels in microvascular beds (Porter et al., 1990), and marks only a few larger vessels (Robertson et al., 2015), thus is often used for microvessel staining to observe and evaluate the microvascular system (Xu et al., 2004; Benton et al., 2008; Maeda et al., 2015).

In conclusion, our findings suggested that 8 min or shorter of ischemia using the suture MCAO method creates an appropriate TIA model in C57BL/6 mice. This model conforms to the definition of human TIA, in which neither permanent neurological deficits nor cerebral infarcts occurred, but neuronal apoptosis was observed. We also confirmed cerebral vasculature abnormalities in TIA mice at submicron resolution for the first time, providing new insights on vasculature-related pathogenesis and potential treatments of TIA. In addition, we found that NBP promoted angiogenesis and improved cerebral microvessels after TIA by increasing the expression of angiogenic growth factors, which may be considered as a candidate drug for the treatment of TIA.

## Data Availability

The original contributions presented in the study are included in the article/Supplementary Material, further inquiries can be directed to the corresponding authors.
