# Supplementary material for: Dl-3-N-Butylphthalide Promotes Angiogenesis in an Optimized Model of Transient Ischemic Attack in C57BL/6 Mice
Source: Front Pharmacol. 2021 Sep 29;12:751397. doi: 10.3389/fphar.2021.751397 (PMC8513739; doi:10.3389/fphar.2021.751397)
Supplement: Supplementary file 1 [file DataSheet2.PDF]

Supplementary Figure 3. Original images of western blot analysis for VEGF/Ang-1/Ang-2 of Figure 6C,D.

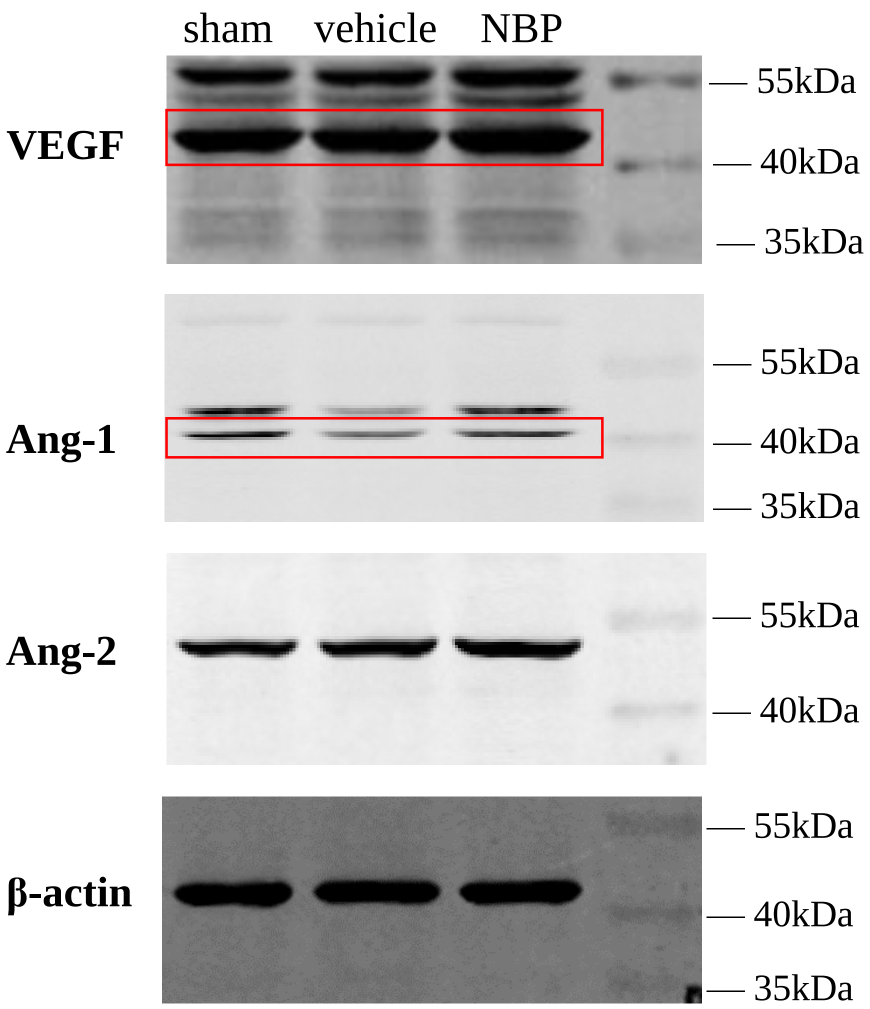

Figure 6C. 7d after MCAO

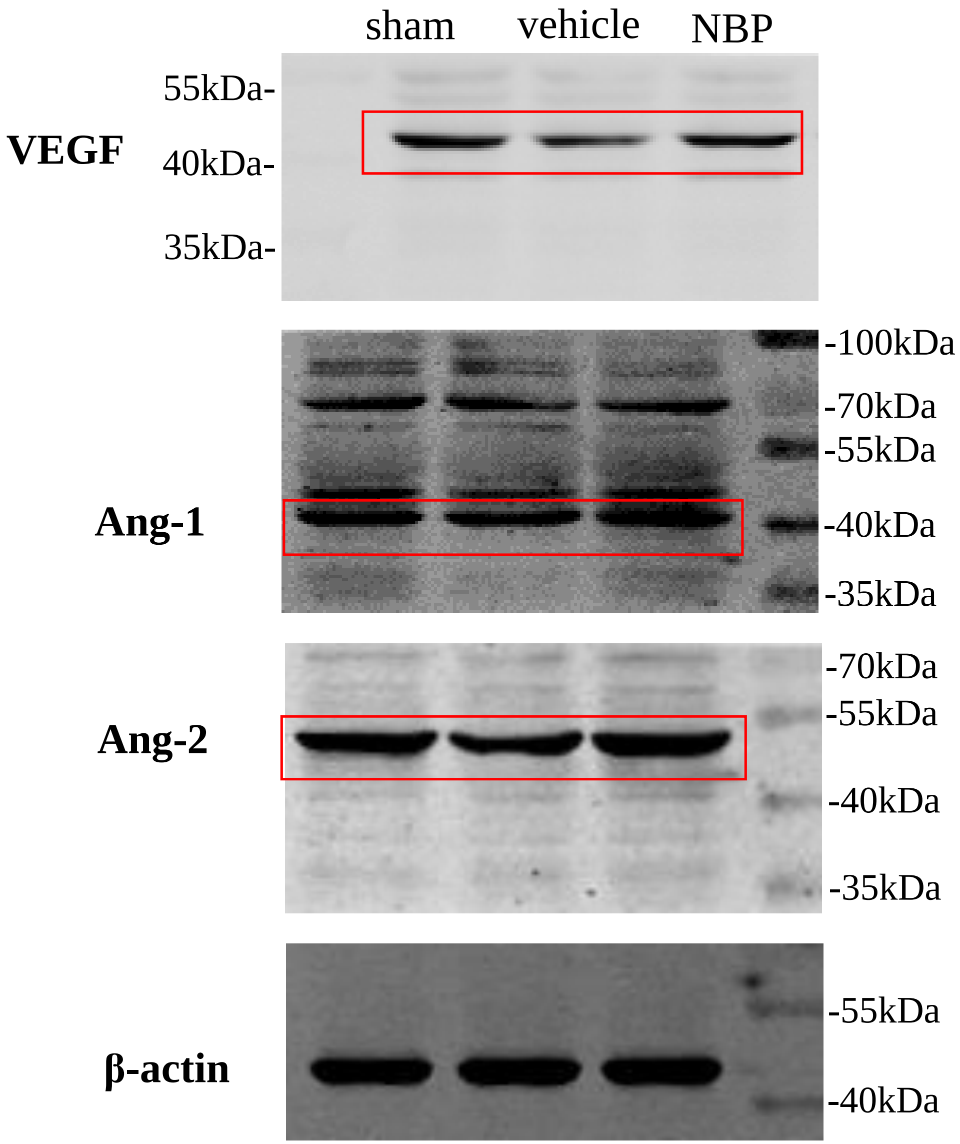

Figure 6D. 14d after MCAO
