# Supplementary material for: Dl-3-N-Butylphthalide Promotes Angiogenesis in an Optimized Model of Transient Ischemic Attack in C57BL/6 Mice
Source: Front Pharmacol. 2021 Sep 29;12:751397. doi: 10.3389/fphar.2021.751397 (PMC8513739; doi:10.3389/fphar.2021.751397)
Supplement: Supplementary file 2 [file DataSheet1.PDF]

Supplementary Material

Supplementary Table 1. Animal groups and number of mice used in the research

Experimental groups	Inclusion		Mortality [*n* (%)]	Exclusion [*n* (%)]	Subtotal (Inclusion+ exclusion)
**Experiment I**					
control		3	0	0	3
7-min		6	0	0	6
8-min		6	0	1*	7
9-min		6	1 (12.50%)	2†	8
10-min		6	0	0	6
**Experiment II**					
LDF-monitor		3	0	0	3
LSCI-monitor		3	0	0	3
**Experiment III**					
**Nissl staining**					
control		3	0	0	3
TIA		4	0	0	4
**Fluorescence staining (CD31, NeuN/TUNEL)**					
control		3	0	0	3
TIA		4	0	0	4
**MOST**					
control		3	0	0	3
TIA		3	0	1*	4
**Experiment IV**					
**Fluorescence staining (CD31)**	**7d**	**14d**			
sham	3	3	0	0	6
TIA + vehicle (vehicle-treated)	3	3	0	0	6
TIA + NBP (NBP-treated)	3	3	1 (14.29%)	1‡	7
**FITC method**	**7d**	**14d**			
sham	3	3	0	0	6
TIA + vehicle (vehicle-treated)	3	3	0	0	6
TIA + NBP (NBP-treated)	3	3	0	0	6
**Western blot**	**7d**	**14d**			
sham	4	4	0	0	8
TIA + vehicle (vehicle-treated)	4	4	2 (20.00%)	2‡	10
TIA + NBP (NBP-treated)	4	4	0	2*	10
**fMOST**					
TIA + vehicle (vehicle-treated)		3	0	0	3
TIA + NBP (NBP-treated)		3	0	0	3
**Total**		119	4 (3.13%)	9 (7.03%)	128

*Excluded due to inadequate CBF decrease (unsuccessful occlusion); †Excluded due to death

(1 mice) and inadequate CBF decrease (1 mice); ‡Excluded due to death.
LDF, laser Doppler flowmeter; LSCI, laser speckle contrast imaging; MOST, micro-optical sectioning tomography; NBP, Dl-3-N-butylphthalide; FITC, fluorescein isothiocyanate; fMOST, fluorescence MOST.

A total of 128 mice were used and a total of 119 mice were available. Additionally, a total of 4 mice were dead due to MCAO surgery, and a total of 5 mice were excluded due to unsuccessful occlusion.

Supplementary Table 2. Quantitative data on the vasculature in the ischemic hemisphere of control and TIA mice

		Vascular length density (m/mm^3)	Fractional vascular volume (%)	Microvascular length density (m/mm^3)	Fractional microvascular volume (%)
Cortex	control	0.60 ± 0.19	2.09 ± 0.75	0.34 ± 0.10	0.54 ± 0.15
	TIA	0.13 ± 0.03****	0.46 ± 0.10****	0.08 ± 0.03****	0.12 ± 0.04****
Striatum	control	0.76 ± 0.12	2.89 ± 0.59	0.38 ± 0.07	0.63 ± 0.11
	TIA	0.32 ± 0.08****	1.35 ± 0.42****	0.15 ± 0.05****	0.23 ± 0.07****
Hippocampus	control	0.64 ± 0.14	2.49 ± 0.97	0.33 ± 0.07	0.46 ± 0.14
	TIA	0.37 ± 0.13***	1.33 ± 0.58**	0.20 ± 0.07**	0.30 ± 0.09*

(n = 3 mice per group; n = 3 data blocks per brain region)

Data were presented as mean ± SD and determined by unpaired t-test. $^{*}P < 0.05$; $^{**}P < 0.01$; $^{***}P < 0.001$; $^{****}P < 0.0001$ control group vs. TIA group.

Supplementary Figures

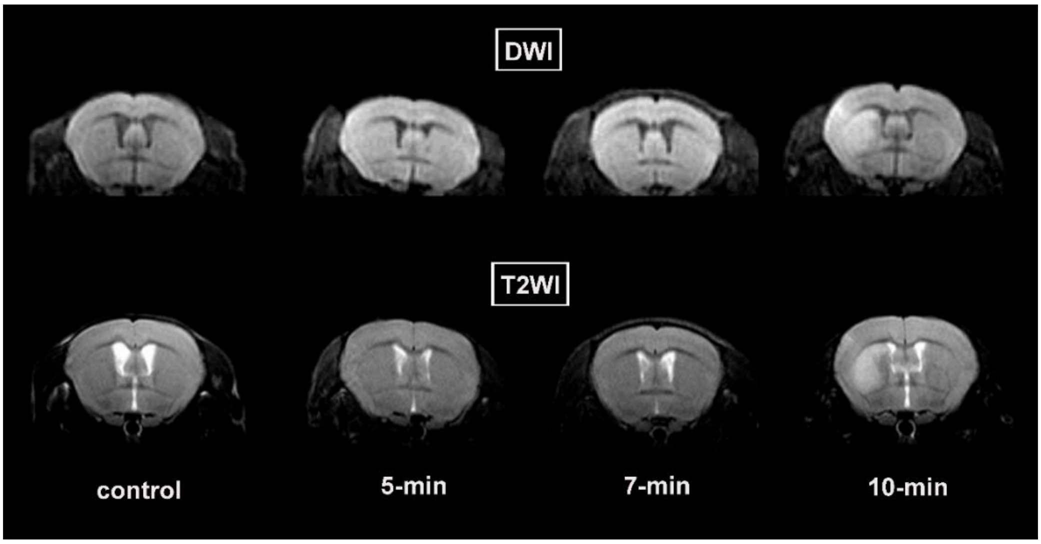

Supplementary Figure 1. Illustrative DWI and T2WI findings in our preliminary experiment. As shown in the figure, 10-min ischemia induced infarcts in the striatum and cortex, but 5- and 7-min ischemia induced no evident MRI abnormalities.

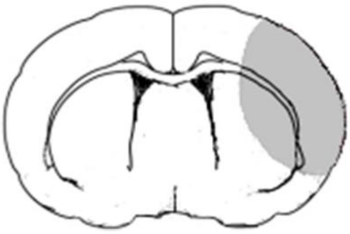

Supplementary Figure 2. A schematic diagram of the images. The gray area represents the area of cerebral ischemia during MCAO surgery, that is, the area of interest in the histological staining and micro-optical sectioning tomography.
